# A novel system of electrodes transparent to ultrasound for simultaneous detection of myoelectric activity and B-mode ultrasound images of skeletal muscles

**DOI:** 10.1152/japplphysiol.00090.2013

**Published:** 2013-08-01

**Authors:** A. Botter, T. M. M. Vieira, I. D. Loram, R. Merletti, E. F. Hodson-Tole

**Affiliations:** ^1^Laboratorio di Ingegneria del Sistema Neuromuscolare, Dipartimento di Elettronica e Telecomunicazioni, Politecnico di Torino, Italy;; ^2^Escola de Educação Física e Desportos, Universidade Federal do Rio de Janeiro, Brazil; and; ^3^Cognitive Motor Function Research Group, Manchester Metropolitan University, Manchester, United Kingdom

**Keywords:** surface EMG, ultrasound imaging, medial gastrocnemius, electrodes, electrical stimulation

## Abstract

Application of two-dimensional surface electrode arrays can provide a means of mapping motor unit action potentials on the skin surface above a muscle. The resulting muscle tissue displacement can be quantified, in a single plane, using ultrasound (US) imaging. Currently, however, it is not possible to simultaneously map spatio-temporal propagation of activation and resulting tissue strain. In this paper, we developed and tested a material that will enable concurrent measurement of two-dimensional surface electromyograms (EMGs) with US images. Specific protocols were designed to test the compatibility of this new electrode material, both with EMG recording and with US analysis. Key results indicate that, for this new electrode material, *1*) the electrode-skin impedance is similar to that of arrays of electrodes reported in literature; *2*) the reflection of US at the electrode-skin interface is negligible; *3*) the likelihood of observing missing contacts, short-circuits, and artifacts in EMGs is not affected by the US probe; *4*) movement of tissues sampled by US can be tracked accurately. We, therefore, conclude this approach will facilitate multimodal imaging of muscle to provide new spatio-temporal information regarding electromechanical function of muscle. This is relevant to basic physiology-biomechanics of active and passive force transmission through and between muscles, of motor unit spatio-temporal activity patterns, of their variation with architecture and task-related function, and of their adaptation with aging, training-exercise-disuse, neurological disease, and injury.

muscle active contribution to motor tasks is traditionally revealed by electromyography (EMG). Since Adrian and Bronk (1) first used concentric needle electrodes to characterize the discharge of nerve fibers during breathing in rabbits, the value of EMG measurements has been recognized in a wide range of fields. The degree and the timing of muscle activation, for instance, might be assessed directly with wire or needle electrodes inserted in the muscle volume, or indirectly from the skin surface (24). Whether intramuscular or surface electrodes should be used depends on the circumstances and on the question one wishes to answer. Because of their larger detection volume, surface electrodes provide a more global view of muscle activation, although at the cost of, occasionally, sampling from fibers belonging to muscles other than those of interest (i.e., cross-talk). In the last decade, technological advances have led to the development of detection methods based on multiple electrode channels overlying either a restricted area of the skin or the entire surface of the muscle. Sampling the two-dimensional potential distribution from multiple locations over the skin surface (i.e., with linear or two-dimensional electrode systems) further reveals anatomical and physiological information, either on the muscle or on the motor unit level (10, 13, 25, 38, 40).

In the past 20 years, medical imaging techniques have been used successfully to quantify anatomical properties of skeletal muscles in vivo. In particular B-mode ultrasound (US) imaging has been used to identify physiologically induced changes in muscle architecture, such as decreases in calf muscle thickness, due to aging (32) and prolonged exposure to weightlessness (6). US imaging can also reveal dynamic changes in fascicle length and orientation caused by changes in joint angle, the degree of muscle activation, or both (16, 30). The recent development of computational, image analysis approaches has revealed that, during postural tasks, there is a very close relationship between activation and sub-millimetric changes in muscle tissue displacement (8, 21). In addition, the high spatial resolution available from US images has also been revealed, with displacements as small as 5 μm measureable (22). US imaging, therefore, has the potential to reveal fine spatial details of tissue displacement, which result from different patterns of activation. Recently, such spatial resolution is beginning to be complemented by increasing temporal resolution, with high-speed imaging (>1,000 frames/s) enabling in vivo estimation of the contractile properties of activated fiber bundles in localized muscle regions (7). Combining synchronous measurements of myoelectric activity and US images would, therefore, provide a novel approach to improving understanding of the association between activation and resulting tissue strain within and across skeletal muscles.

Recent work simultaneously quantifying muscle movement and activation has been limited to sampling US images and surface EMGs from different muscle regions. Typically, surface electrodes and US probes are placed in close proximity to each other (21, 31). For small muscles, such as the intrinsic hand muscles or forearm muscles, this arrangement between US probes and electrodes is overtly not viable. For larger muscles, there might be a sufficiently large skin area for surface electrodes and US probes to sample from different portions of the same muscle. A number of factors are, however, likely to influence how closely EMGs and US images collected from different muscle regions are associated. Specifically, there are a growing number of reports detailing regional patterns of muscle activation, even in muscles with a single line of action (e.g., ankle extensors; Refs. 15, 17, 39, 42, 43, 46). In addition, many muscles in the lower limb have distinct regional variation in fascicle architecture (36, 45) and aponeurosis properties (28) and behavior (18, 37). Our proposed grid of electrodes transparent to US would, for the first time, enable fuller, simultaneous regional analysis of muscle activation and resulting mechanical tissue displacement. New insights could be gained into force transmission and into the interaction between active and passive tissue components within the muscle. Furthermore, if combined with high-frame rate (1,000–5,000 frames/s) US machines (e.g., 7), grids of electrodes transparent to US would allow us to study the spatial and temporal propagation of muscle tissue displacements related to different patterns of muscle activation (e.g., involuntary twitches such as fasciculations). Physiologically, this would open a new window into the analysis of motor unit activity and associated muscle tissue mechanics relevant to studying neurodegenerative diseases, injuries (e.g., nerve crush), and aging.

Sampling US images and surface EMG from the same muscle region requires the US probe to lie over the electrodes. In this case, the view of muscle tissue could be hindered by the acoustic impedance of electrodes and their supporting material. Indeed, as shown in Fig. 1, the muscle tissue cannot be observed in images collected with the US probe positioned over different arrays of electrodes proposed in literature (hereafter defined as conventional). In this study, we, therefore, designed and tested a new system for the detection of surface EMGs transparent to US (US-EMG electrodes). Specifically, by combining two layers of silicon rubber, we were able to create surface, gelled electrodes that produce negligible echo in US images. The compatibility of this new electrode material, both with EMG recording and US analysis, was tested with in vivo and in vitro protocols.

**Fig. 1.**
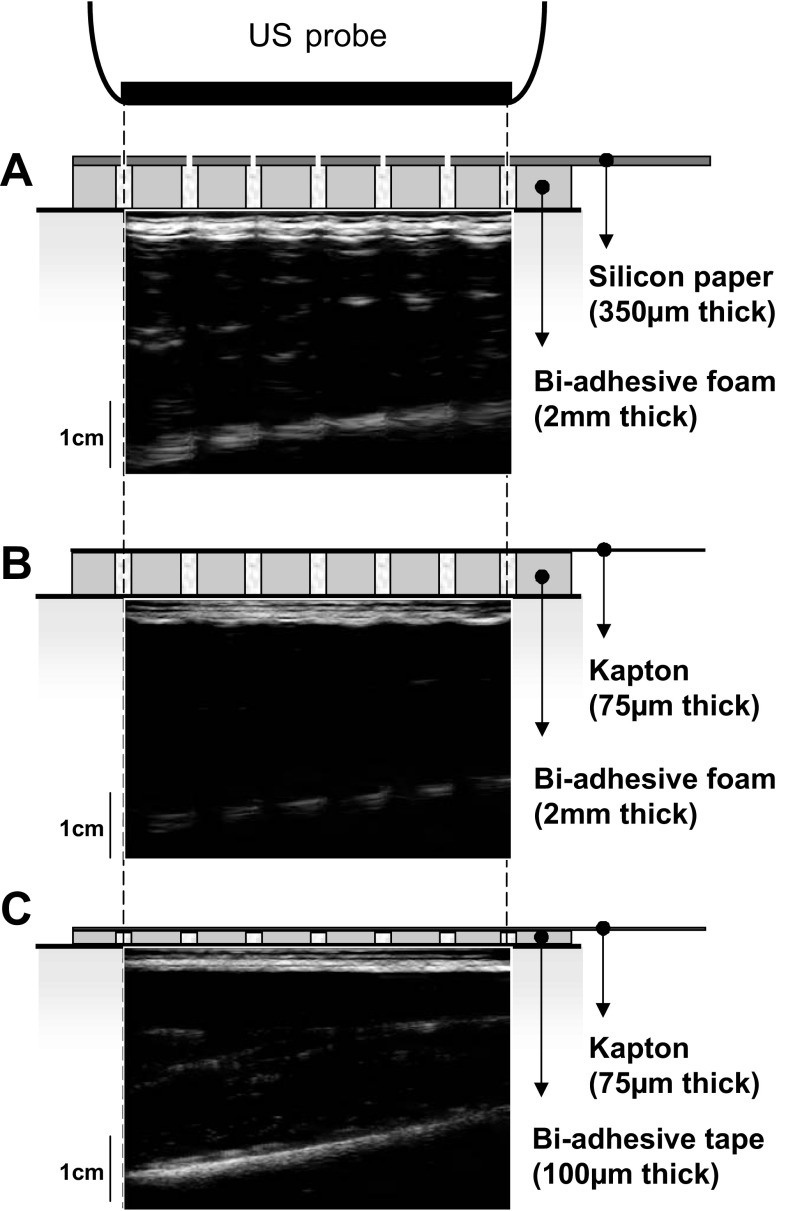
Ultrasound (US) images collected with different arrays of electrodes. US images collected with the US probe positioned over different configurations of electrode arrays reported in literature are shown. Arrays are constituted by a carrier material, which includes electrodes and their connections. Electrical contact between electrodes and skin is ensured by injecting conductive gel in the cavities of a biadhesive material interposed between arrays and skin. The three panels show US images collected with three different combinations of carrier and biadhesive material: silicon-coated paper and biadhesive foam (*A*), flexible printed circuit (kapton) fixed to the skin with biadhesive foam (*B*), and tape (*C*). Electrodes interposed between the US probe and the skin fully hinder the view of muscle tissue. *A*: for the configuration shown on *top*, conductive gel between electrodes and skin is injected through gaps present in the carrier material in correspondence of the electrodes. Note that, in this case, the US gel interposed between the US probe and skin would likely lead to short circuits between holes of neighboring electrodes.

## METHODS

### Electrode Grid Design

**Fig. 2.**
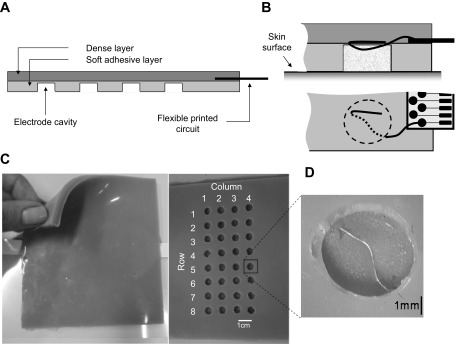
The US-EMG system of electrodes. *A*: schematic representation of a longitudinal section of the dense and the soft silicon layers. The layer interfacing the skin (light shaded) is soft and adhesive and houses circular cavities where wire electrodes are exposed (detailed in *B* and *D*). The external layer (dark shaded) is dense and provides sufficiently rigid support for the printed circuits connecting the electrodes to the amplifier. Pictures of the dense layer, of the adhesive layer housing electrodes, and of a single cavity containing an exposed wire electrode are shown in *C* and *D*.

Electrodes were embedded into a layer of silicon rubber (Elite Double 8, Zhermack Spa, Badia Polesine, Italy). The US-EMG system of electrodes is composed of two superimposed layers (Fig. 2*A*): *1*) the layer interfacing the skin is soft and adhesive, to avoid the formation of air bubbles between it and the skin; and *2*) the second layer is denser and houses the electrical connections between the electrodes and the amplifier.

Each electrode consists of a stainless steel wire (0.1 mm diameter) exposed within a small, circular cavity (4 mm diameter) in the adhesive silicon layer (Fig. 2, *B* and *D*). In the current configuration, the grid is made of 32 cavities (electrodes) arranged in 4 columns and 8 rows, with 10-mm distance between adjacent electrodes (Fig. 2*C*). Electrical contact between wires and skin is ensured by filling each cavity with conductive paste (TEN 20 Conductive Paste, Weaver). Wire electrodes are connected to the amplifier through two flexible printed circuits (i.e., one for each group of 16 electrodes), which are partially fixed into the dense layer and placed a few centimeters from the first and fourth columns of electrodes. Printed circuits are obtained with photolithographic technology, with paths of conductive material deposited on a flexible plastic substrate (Kapton polyimide film, 100 μm thick). Each of the two printed circuits connects a group of 16 wires to an external, standard connector (ZIF 16 ways, 1-mm pitch, Tyco Electronics).

### Experimental Protocols: Testing US-EMG Electrodes

Specific protocols were designed to test how much the quality of surface EMGs and US images change when they are collected with our US-EMG system of electrodes. Details on these protocols are given in the following sections. Briefly, we carried out three studies to investigate the following: *1*) the noise and the impedance of the electrode skin interface; *2*) the influence of the US-EMG electrodes on the US-based estimation of movements imposed on a spring; and *3*) the effect of the US-EMG electrodes on the quality of US images and surface EMGs recorded simultaneously from the medial gastrocnemius (MG) muscle during electrically evoked twitches.

#### Electrical characterization of the electrode-skin interface.

The properties of the electrode-skin interface can strongly affect the quality of the detected EMGs. Ensuring low and balanced impedances across electrode-skin interfaces is critical for the detection of high quality EMGs, thus minimizing artefacts and external sources of interference (23). In this study, therefore, we quantified and compared the electrode-skin noise and the impedance of our US-EMG system with those of commercially available electrodes with similar electrode-skin contact surface (semidisposable adhesive arrays, four electrodes, 10-mm interelectrode distance, Spes Medica, Battipaglia, Italy). We selected this type of electrode because the size of the contact area between the conductive paste and the skin is similar (10 mm^2^) to that of our US-EMG electrodes (12.5 mm^2^). Impedance and noise measurements were taken from the ventral portion of the forearm of 10 male subjects (mean ± SD; age: 28.1 ± 3.9 yr; height: 177.8 ± 6.5 cm; body mass: 75.6 ± 9.3 kg). Before participating in the study, subjects received a detailed explanation of the protocol and provided written, informed consent. The study conformed to the guidelines in the Declaration of Helsinki and was approved by the local Ethics Committee. A pair of US-EMG electrodes and a pair of conventional electrodes were positioned, alongside each other, over a skin region cleaned with water. To assure adequate electrode-skin contact, a conductive paste (TEN 20 Conductive Paste, Weaver) was inserted into the cavities of the two electrode types. Noise and impedance measurements were taken between the two electrodes of each pair.

The noise of the electrode-skin interface was measured with a custom-made noise amplifier [Laboratorio di Ingegneria del Sistema Neuromuscolare (LISiN), Politecnico di Torino, Italy] with the following characteristics: 12,000 amplification; 100-dB common mode rejection ratio; 100 GΩ//2 pF input impedance; 0.8 μV referred-to-input noise in the 2.5- to 1,000-Hz band; 0.1–1,000 Hz bandwidth. The output of the noise amplifier was digitized with a National Instrument data acquisition device (USB-6210, sampling frequency: 10 kHz, resolution of the A/D converter: 16 bits) and then band-pass filtered (10–1,000 Hz, second-order Butterworth filter). During acquisition, subjects were asked to relax as much as possible to avoid including motor unit action potentials in the noise measurements. The absence of motor unit action potentials, power line interference, and movement artifacts in the detected signal was ensured by visual inspection. Root mean square (RMS) amplitude of the recorded signal was computed over the entire acquisition (10 s). The electrode-skin interface and the amplifier were the likely sources of noise contributing to such RMS amplitude. Assuming these two sources are independent, the RMS noise due to the electrode-skin interface was estimated by quadratic subtraction of the amplifier RMS noise (0.8 μV; see above) from the measured RMS amplitude.

The impedance of the electrode-skin interface was measured with a custom-made impedance meter (LISiN, Politecnico di Torino, Italy). This device converts a sinusoidal voltage input into a proportional current signal (200 nA peak-to-peak amplitude) and measures the voltage drop between the electrodes tested. Both the sine wave, used to drive the current signal, and the voltage drop were sampled with the same data acquisition device used for the noise measurements. The magnitude and the phase of the impedance were quantified as the amplitude ratio and the phase shift between the measured voltage and injected current, for a frequency sweep in the range 10–1,000 Hz.

#### In vitro testing of the US-EMG electrodes.

**Fig. 3.**
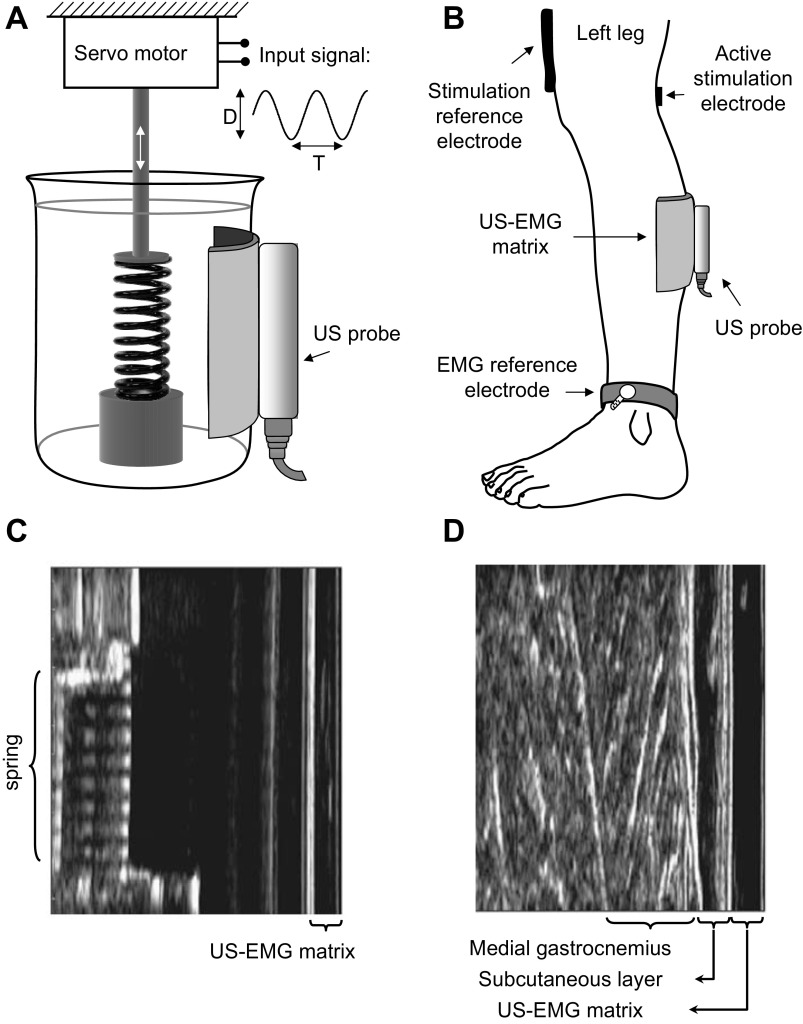
Protocols used for testing the US-EMG system of electrodes. *A*: schematic representation of the experimental setup considered for the in vitro testing of the US-EMG electrodes. US images were recorded to estimate movement of a spring immersed in a beaker filled with water. A motorized device applied sinusoidal changes to the spring length with regular amplitudes (D: 0.4–1.4 mm) and frequencies (1/T: 0.2–10 Hz). *B*: schematic representation of the experimental setup adopted for the in vivo testing of the US-EMG system of electrodes. The active stimulation electrode (cathode, 10 × 10 mm) was positioned in correspondence to the tibial nerve branch supplying the gastrocnemius muscle, whereas the stimulation reference electrode (damp cloth anode, 80 × 50 mm) was placed just above the patella. Three sessions (trials) of electrical stimulation were carried out. Surface EMGs were detected with the US-EMG electrodes (see Fig. 2) during the first and second trials. Ultrasound images were collected by positioning the US probe over the US-EMG electrodes in *trial 2* and on the skin surface in *trial 3*. US images collected during the experimental protocols illustrated in *A* and *B* are shown, respectively, in *C* and *D*.

We tested whether calculations of the oscillating length of a spring, determined from sequences of US images, were influenced by the presence of the US-EMG material. One end of the spring was weighted to the base of a plastic beaker. The other end was secured to a rigid metal arm, fixed to a platform that could be rotated using a custom-built, servomotor (Fig. 3*A*). A layer of acoustic gel was applied to a linear US probe (7 MHz, 59 mm field of view, LogicScan 128, Telemed, Vilnius, Lithuania), which was secured to the side of the beaker using an elasticized bandage. The beaker was filled with water, and the US settings adjusted to ensure good image quality.

The spring was driven through constant-amplitude oscillations over a 30-s period using custom-written MatLab and SimuLink scripts (The MathWorks, Natick, MA), while US images (80 Hz) were collected and saved to a dedicated laptop computer. Twenty-seven conditions were tested, comprising three peak-to-peak amplitudes (0.4, 1.0, and 1.4 mm) repeated over nine oscillation frequencies (0.5, 0.7, 1, 2, 3, 5, 7, 8, and 10 Hz), chosen to reflect potential changes in MG length during postural control. Once all conditions had been recorded, the US probe was repositioned with a sample of the US-EMG material added to form an interface between it and the beaker. All 27 oscillation conditions were then repeated. US images and the length changes imposed to the spring (i.e., the output of the driving motor) were recorded synchronously.

#### In vivo testing of the US-EMG electrodes.

When surface EMGs and US images are acquired from the same portion of muscle, the two detection systems could interfere with each other. An experimental protocol was designed to test the effect of our US-EMG system on the US images and surface EMGs (Fig. 3*B*). These data were collected at Manchester Metropolitan University, where the experimental protocol was approved by the Academic Ethics Committee of the Faculty of Science and Engineering. All participants gave written, informed consent to participate in the experiments, which conformed to the standards set by the latest revision of the Declaration of Helsinki.

Five male and one female volunteers (mean ± SD; age: 36.3 ± 6.8 yr; height: 176.7 ± 10.5 cm; body mass: 72.0 ± 7.8 kg) stood on custom-built footplates and were strapped about their waist to a fixed board, enabling them to stand without swaying and with no/minimal activation of the ankle extensor muscles. The skin over the MG muscle of the left leg was shaved and cleaned. Compound muscle action potentials were elicited using electrical stimulation (Digitimer DS7, Digitimer, Welwyn Garden City, UK). A large anode electrode (80 × 50 mm, damp cloth) was fixed to the leg, just above the patella, using elasticized bandage (Vetrap, 3M United Kingdom PLC, Bracknell, UK). The skin location closest to the branch of the tibial nerve supplying the MG muscle was identified by scanning the popliteal region with a stimulation pen electrode (small size cathode: 1 cm^2^ surface; Globus Italia, Codognè, Italy). Biphasic current pulses (square wave, 200-μs duration) were delivered at increasing current levels, with a frequency of 2 pps; the selected stimulation site corresponded to that where an electrical pulse evoked a muscle twitch with the least injected current. Thereafter, the cathode (circular, pregelled electrode 10 mm diameter) was positioned over the selected skin location.

M-waves and associated movements of the MG muscle were recorded with the US-EMG grid of electrodes positioned at a predetermined skin region. Initially, the skin location where low-level stimulation led to the representation of strongest M-waves was identified using a 16 Ag-bar linear electrode array (10-mm interelectrode distance; LISiN, Politecnico di Torino, Italy) and marked on the skin. The stimulation level was set by identifying the lowest amplitude at which a clear M-wave occurred, accompanied by visible movement in the US image (stimulation amplitude; mean ± SD: 9.33 ± 2.66 mA). The columns of the electrode grid were then aligned along the muscle proximal-distal direction, with the region identified by the linear array being located between the second and the third columns.

The experimental protocol consisted of three trials. First, the US-EMG electrodes were placed on the leg over the marked region. M-waves were then recorded as the muscle was stimulated at 1 pps for 20 s with biphasic current pulses (square wave, 200-μs duration) at the amplitude defined in the pretesting session. Surface EMGs were acquired in monopolar configuration with respect to a reference electrode placed at the ankle. EMGs were amplified (EMG-USB amplifier, OT Bioelettronica, Torino, Italy), band-pass filtered (10–750 Hz, 3-dB bandwidth), sampled at 2,048 samples/s (12-bit A/D converter), displayed in real time, and stored on a disk of a personal computer. To synchronize the stimulation and the M-wave acquisition, the trigger signal of the stimulator was sampled at 2,048 samples/s and stored.

In the second trial, the US probe was covered with a layer of acoustic gel and secured using elasticized bandage over the top of the matrix in correspondence of the marked region (between *columns 2* and *3* of the US-EMG matrix). A further 20 s of electrical stimulation, at the same amplitude and frequency as in the first trial, was then completed, while myoelectric data and US images (80 Hz) were collected on dedicated laptop computers, synchronized using a common trigger.

In the third trial, US images were sampled during the same stimulation protocol adopted in *trials 1* and *2* by placing the US probe directly on the skin surface without interposition of the US-EMG electrodes. The US probe was placed on the leg over the marked region defined in the pretesting session.

By comparing the M-wave detected in *trials 1* and *2*, we tested whether the presence of the US probe over the top of the matrix affected the quality of the detected surface EMGs. The influence of the US-EMG electrodes on US images was investigated by comparing the US-based predictions of gastrocnemius length change during electrically evoked twitches in *trials 2* and *3*. The test was performed during electrically induced contractions to have, for each subject, a stable pool of active motor units during the entire protocol and, therefore, a stable set of mechanical twitches and M-waves to be compared between the different experimental conditions.

### US Image Analysis: Influence of the US-EMG Electrodes on B-mode US Imaging

#### Calculation of image intensity values.

To determine whether the presence of the US-EMG electrodes altered image characteristics, image intensity histograms were compared. If the presence of the US-EMG electrodes introduced additional noise (e.g., salt and pepper features), the distribution of the intensity histograms would be expected to differ between conditions [e.g., increased higher intensity (whiter) values]. All images were analyzed using custom-written LabVIEW code (LabVIEW 2009, National Instruments, Austin, TX). In each image, a standardized region of interest (ROI) was defined (599 × 438 pixels for images of the spring; 380 × 400 for muscle images). In all cases, the ROI was chosen to remove the matrix material itself from the analysis (e.g., starting just below the reflection from the container or skin surface) and to ensure similar muscle features were represented across all participants. This ensured that exactly the same features were present in all analyses (i.e., the echo of the matrix material itself did not influence results). Mean pixel intensity values were calculated for each frame of each trial, and mean values were calculated across trials and conditions. The average intensity histogram, identifying the mean number of pixels at each intensity, was also calculated for each condition.

#### Calculation of spring oscillations.

In a representative image of each trial, eight 10 × 15 pixel ROIs were identified to define the longitudinal edges at each end of the spring. The position of each ROI was then tracked through all images of the trial using spatial cross-correlation (22). This approach was used as it is an excellent means of tracking small movements, which can be readily applied to any imaged object. In each tracked image, the median position of the central pixel of the ROIs at each end of the spring was calculated, and the distance between them provided a measure of spring length. Pixel values were converted to millimeter distances using machine/trial-specific calibration details. The normalized mean square error (NMSE) between predicted and known changes in spring length was determined for each trial according to *Eq. 1*, where *N* is the number of samples (2,400 samples corresponding to 30 s at 80 Hz); *f*(*n*) is the predicted change in spring length (estimated with or without the presence of the US-EMG material between the US probe and the plastic beaker); and *g*(*n*) is the change in spring length imposed by the servomotor.

(1)) $$NMSE=100\cdot \frac{\overset{N}{\underset{n=1}{\sum }}{\left[f(n)-g(n)\right]}^{2}}{\overset{N}{\underset{n=1}{\sum }}{\left[g\;(n)\right]}^{2}}$$

#### Calculation of muscle twitch characteristics.

Following the methods of Darby et al. (5), a velocity field representation of local movement was found within each collected image sequence by identifying and tracking distinctive and persistent features using a Kanade-Lucas-Tomasi (KLT) feature tracker (2). The velocity field was subdivided into aponeurosis and fascicle regions using segmentations from a multiresolution active shape model (ASM). As KLT features are automatically identified and tracked, the approach results in collections of the “best” features being identified in each image frame. This removes the requirement for individual features to persist and be identifiable in all images of a sequence, but means that tissue movement must be estimated based on the collection of KLT features, which come and go throughout the image sequence. To do this, a set of measurement probes, arranged in an 8 × 10 grid, are placed on the image at the initialization frame. Placement is fully automated and based on the ASM segmentation, such that the outer rows lie on the deep/superficial aponeurosis, respectively, and the remainder are equally distributed across the fascicle region. Movement of each probe was determined by the movement of all persisting KLT feature tracked on the ASM segment defined by the probe location (e.g., deep/superficial aponeurosis or fascicle region) using triangle-based linear interpolation (5).

MG twitches from each trial were characterized by calculating the mean distance between measurement probes located in the deep and superficial area of the fascicle region in all collected images. Thickness of the muscle in each frame was calculated as the distance between the inner ASM segmentation boundaries of the deep and superficial aponeuroses. Pixel values were converted to millimeter distances using the machine/trial-specific calibrations. Length and thickness changes predicted from images collected with and without the US-EMG interface were aligned using cross-correlation and compared with correlation and model II regression analysis. We selected this approach for analysis of muscle twitches as it provides a robust means of tracking larger tissue movements, which has been specifically developed for assessment of skeletal muscle.

#### Influence of the US probe on surface EMGs detected with US-EMG electrodes.

Monopolar surface EMGs collected during low-level electrical stimulation of the tibial nerve were initially band-pass filtered (20–450 Hz, 4th-order noncausal Butterworth filter). For each electrode of the US-EMG grid, M-wave templates were calculated as the average of 20 consecutive M-waves (40 ms from each rising edge of the stimulation trigger). For each trial, then, one set of 32 averaged M-waves was obtained. To investigate possible interference from the US probe on the surface EMGs (Fig. 3*B*, *trial 1* vs. *trial 2*), the NMSE was calculated for each of the 32 pairs of M-waves detected with and without the US probe positioned on the US-EMG electrodes. With reference to *Eq. 1*, *N* is now the number of samples of each M-wave template (*N* = 82 samples, which amount to 40 ms); *f*(*n*) is the M-wave template calculated by averaging all 20 M-waves detected while the US probe was applied over the US-EMG matrix (*trial 2*); *g*_*ij*_(*n*) is the M-wave template obtained without the US probe lying over the US-EMG electrodes (*trial 1*).

The effect of positioning the US probe on EMG waveforms was compared between different pairs of columns of electrodes. As greater occurrence of bad channels or amplitude alterations was expected for EMGs recorded by the columns of electrodes immediately below the US probe, the NMSE over the matrix was grouped according to the columns above which the US probe was positioned. Error values averaged across the two central columns (see Fig. 7*A*: *columns 2* and *3*) were compared with those averaged across the two lateral columns (Fig. 7*A*: *columns 1* and *4*).

### Statistical Analysis

Electrode-skin impedance measured at different frequencies with the US-EMG electrodes was compared with that measured with conventional electrodes using a two-way analysis of variance. Paired *t*-tests were used to compare the noise amplitude at the electrode-skin interface. Similarly, the null hypothesis that the waveform of M-waves detected with and without US probe lying over the US-EMG electrodes was not different between central and lateral columns of electrodes was tested with paired *t-test.* The mean image intensity between conditions in the spring oscillations was compared using *t*-test and, due to the smaller sample size, using Wilcoxon signed-rank test for recordings of muscle twitches. The potential differences in the mean distribution of pixel intensities (intensity histograms) were investigated using Kolomogorov-Smirnov test. Differences in the mean square error calculated for spring oscillations at different conditions were compared with analysis of variance (3 amplitudes × 9 frequencies × 2 detection conditions, with and without the US matrix). Similarities in the shape and amplitude of gastrocnemius twitches quantified from US images collected with and without the US-EMG electrodes interposed between the US probe and the skin were tested with Pearson correlation and model II regression analysis. In all cases, significance level was set at 5%, and data are reported as means ± SD.

## RESULTS

### Electrode-skin Impedance and Noise

**Fig. 4.**
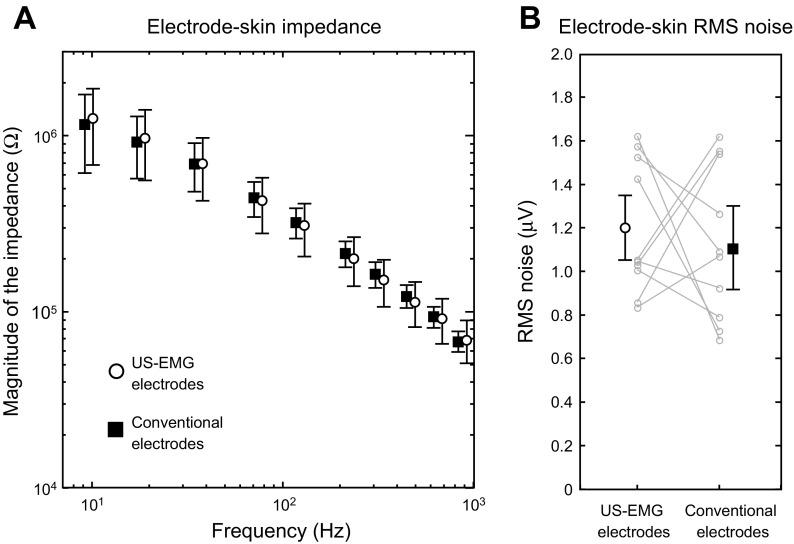
Electrical characterization of the electrode-skin interface. *A*: magnitude of electrode-skin impedance measured between a pair of US-EMG (○) and conventional (■) electrodes (1 cm apart) within the 10-Hz to 1-kHz frequency range. *B*: root mean square (RMS) noise of the electrode-skin interface for the US-EMG and conventional electrodes. Noise values from individual subjects are displayed in light shading.

Electrical characteristics of US-EMG and conventional electrodes were similar. Regardless of the frequency tested, from 10 to 1,000 Hz, no significant differences were observed for the electrode-skin impedance between the US-EMG electrodes and the conventional electrodes (Fig. 4*A*; *P* = 0.47; *N* = 10 subjects × 10 frequencies). With respect to the noise of the electrode skin interface, RMS noise amplitude was comparable between the two systems (US-EMG electrodes: 1.2 ± 0.31 μV; conventional electrodes: 1.1 ± 0.36 μV; *P* = 0.69; *N* = 10 subjects; Fig. 4*B*).

### Effects of the US-EMG System of Electrodes on the US Images

Visual inspection of US images revealed that the silicon material constituting the US-EMG electrodes had marginal effect on the representation of tissue features. The chief difference introduced by the use of the silicon matrix was the additional layer clearly seen at the top of the image (Fig. 3, *C* and *D*). In several instances, when the US probe was lying exactly above the second or third column of electrodes, small echoes from the conductive paste could be seen as shadows going deep through the image. These were overtly most predominant at image regions closest to the skin, where shadow thickness was largest. Such shadowing was actually found to be useful for identifying the exact location of the US probe in relation to the electrode channels and did not affect the representation of muscle features. *T*-test comparison of the mean pixel intensities between images of the spring collected with and without the US-EMG electrodes showed a significant difference (*P* < 0.001) in pixel intensity when the electrodes were in place (35.18 ± 0.30 with electrodes vs. 30.62 ± 0.41, with 0 = black and 255 = white). In contrast, Wilcoxon signed-rank test revealed there was no significant difference in mean pixel intensities between images of MG collected with and without the US-EMG electrodes (*P* = 0.295, 64.04 ± 15.48 with electrodes vs. 65.85 ± 13.84). The distribution of pixel intensities between conditions did not differ significantly in either spring or muscle conditions (Kolomogorov-Smirnov test: *P* = 0.719 for spring; *P* = 0.759 for muscle), indicating that the presence of the US-EMG electrodes did not significantly alter image characteristics.

**Fig. 5.**
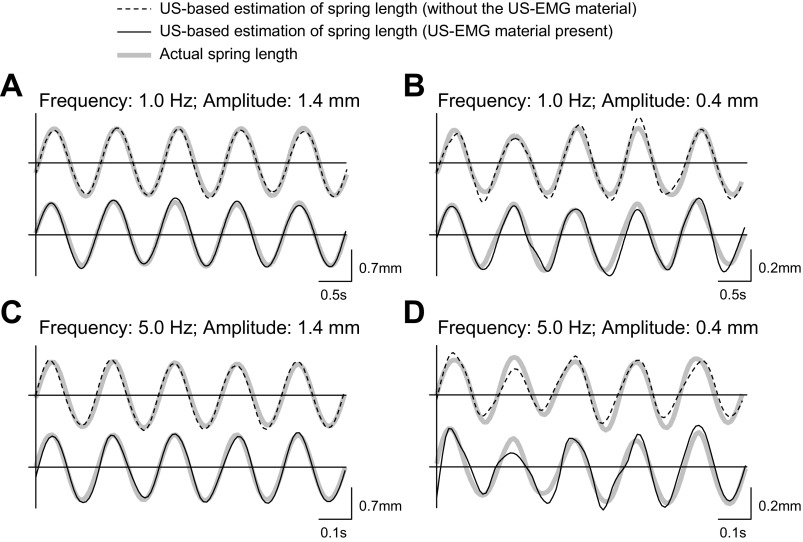
Effect of the US-EMG material on US-based estimation of spring length changes. Spring length changes estimated from US images collected with and without the US-EMG material positioned between the US probe and the plastic beaker are shown. Representative length estimations from four combinations of oscillation frequency and peak-to-peak amplitude are shown: frequency = 1.0 Hz, amplitude = 1.4 mm (*A*); frequency = 1.0 Hz, amplitude = 0.4 mm (*B*); frequency = 5.0 Hz, amplitude = 1.4 mm (*C*); frequency = 5.0 Hz, amplitude = 0.4 mm (*D*). For each oscillation condition, the actual spring length (shaded traces) and the US-based estimation of spring length obtained without (dashed traces) and with (solid traces) the US-EMG material are shown.

Example traces from four of the spring oscillation conditions are shown in Fig. 5. Close inspection of Fig. 5 reveals that estimation errors tended to be larger for imposed oscillations of smaller amplitudes (see *A* and *B*, and *C* and *D*, in Fig. 5) or of higher frequencies (see *A* and *C*, and *B* and *D*, in Fig. 5). Indeed, the three-way analysis of variance showed that the NMSE computed between predicted and imposed changes in spring length (Table 1) was significantly affected by oscillation frequency (main effect; *P* < 0.001, *F* = 156.6; *N* = 3 amplitudes × 2 conditions) and amplitude (main effect; *P* < 0.001, *F* = 1,423; *N* = 9 frequencies × 2 conditions). On the other hand, regardless of the frequency and amplitude tested, imposed spring length changes were estimated equally well from US images collected with and without the US-EMG material interposed between the US probe and the plastic beaker (*P* = 0.96, *F* = 0.002; *N* = 3 amplitude × 9 frequencies). These results demonstrate the agreement between spring lengths calculated from images collected with and without the US-EMG material present.

**Table 1.** NMSE (Eq. 1) between the estimated and the actual spring length change for two detection conditions: with and without the ultrasound-electromyogram material between the ultrasound probe and the plastic beaker

	Oscillation Peak-to-Peak Amplitude, mm		
Oscillation Frequency, Hz	0.4	1.0	1.4
0.5			
Without	5.0	1.8	1.5
With	6.3	2.2	1.6
0.7			
Without	5.8	1.3	1.1
With	8.2	2.0	1.3
1.0			
Without	7.6	2.1	1.5
With	9.6	2.5	1.7
2.0			
Without	15.3	3.9	2.4
With	14.1	3.6	2.3
3.0			
Without	17.0	4.6	2.3
With	19.9	4.4	2.3
5.0			
Without	24.3	5.9	2.6
With	22.9	5.3	3.4
7.0			
Without	30.7	5.7	3.7
With	31.6	6.7	2.4
8.0			
Without	34.8	6.5	3.7
With	31.7	7.3	3.3
10.0			
Without	43.1	8.7	8.5
With	39.4	9.2	3.7

Values are normalized mean square error (NMSE) with and without the ultrasound-electromyogram material (in %).

**Fig. 6.**
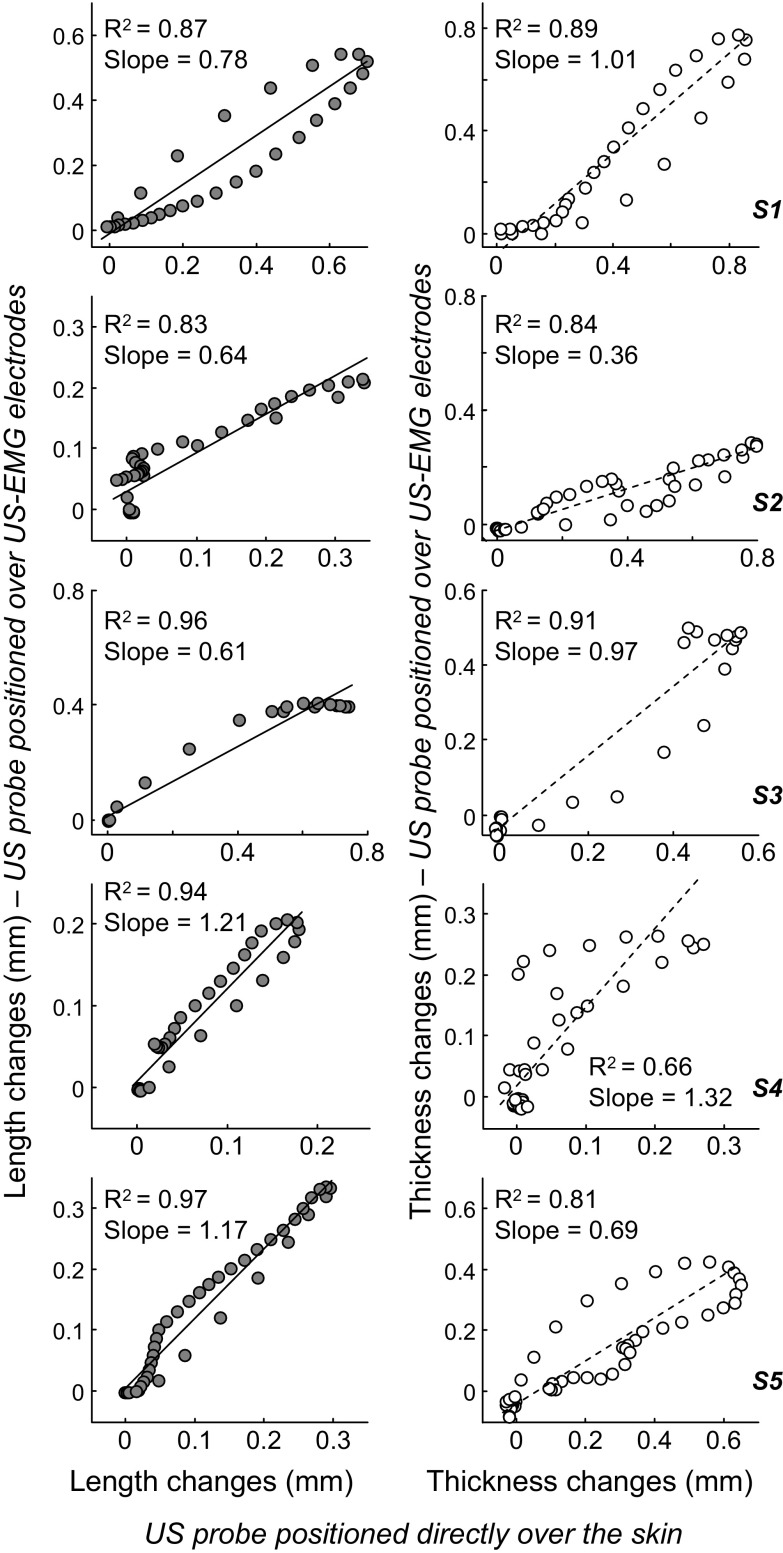
Effect of the US-EMG electrodes on electrically elicited changes in muscle shape. Scatter plots created from twitches averaged across 10 stimuli are shown for each participant. Values obtained with the US probe positioned over our US-EMG electrodes are shown in the ordinate, whereas those obtained with the US probe lying over the skin are shown in the abscissa. Estimated changes in medial gastrocnemius length and thickness are shown in the *left* column (shaded circles) and *right* column (open circle), respectively. Pearson coefficient of determination (*R*^2^), and the slope of the regression lines are reported within each scatter plot. Data from one participant were excluded from this analysis because the trigger signal was not sampled with the US images.

Similar to the in vitro results, the electrically elicited twitches (gastrocnemius thickness and length) estimated from US images collected with and without the silicon matrix exhibited similar profiles. Such a similarity led to statistically significant correlations between twitches (Fig. 6). With reference to the values reported in Fig. 6, the unit slope of the regression line would indicate equal amounts of movement of gastrocnemius fascicles in both conditions. Some participants showed regression lines whose slope was markedly close to unit (length estimates: *subject 5*; thickness estimates: *subjects 1* and *3*). Other participants, however, showed either lesser or greater amounts of fascicle movement in US images collected with, rather than without, the US-EMG matrix. Notwithstanding these differences in amplitude across participants, there was a consistent, statistically significant association between gastrocnemius movements estimated from images recorded with and without the matrix (Pearson *R*^2^ > 0.83 for length estimates; *R*^2^ > 0.66 for thickness estimates; *P* < 0.001 for all cases).

### Effect of the US Probe on M-waves Collected with the US-EMG Electrodes

**Fig. 7.**
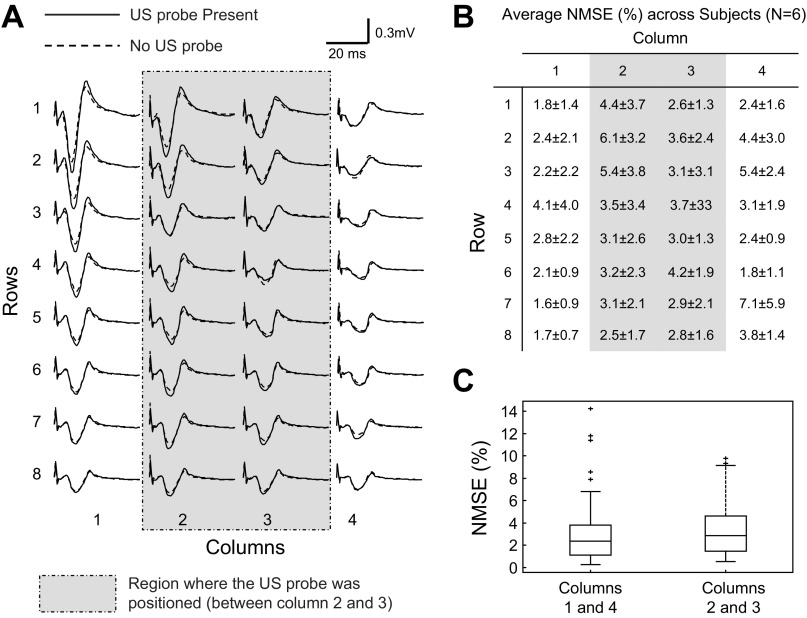
Effect of the US probe on M-wave waveform. *A*: monopolar M-waves detected from the 32 electrodes of the ultrasound matrix in one representative subject. For each electrode, two signals are reported: M-wave detected with (*trial 2*, solid line) and without (*trial 1*, dashed line) the US probe lying over the matrix. The shaded rectangle represents the region within which the US probe was positioned (between *columns 2* and *3*). *B*: average normalized mean square error (NMSE) between M-waves acquired with and without ultrasound probe lying over US-EMG electrodes (see Fig. 2). For each channel, NMSE was computed according to *Eq. 1* and averaged across six subjects. *C*: NMSE values for the lateral (*1* and *4*) and central (*2* and *3*) columns of electrodes are shown (*N* = 6 subjects × 16 electrodes × 2 pairs of columns), with *columns 2* and *3* being closest to the US probe. Horizontal lines denote the median values, whereas boxes indicate the interquartile intervals and whiskers correspond to the non-outlier intervals.

Electrically elicited potentials were represented equally well in surface EMG detected with and without the US probe lying over the US-EMG electrodes. Figure 7*A*, for example, shows the monopolar M-waves detected from one representative subject in the two experimental conditions. Visual inspection of Fig. 7*A* reveals that M-waves have similar waveforms, regardless of whether detected in the central columns (*columns 2* and *3*), where the US probe was positioned, or the lateral columns of electrodes. Indeed, when considering all participants, the average value for the NMSE between M-waves detected in both conditions was markedly low (3.3 ± 2.6%; global average across all 32 electrodes; *N* = 6 subjects; see Fig. 7*B*). The average error between M-waves in the central columns of electrodes (3.6 ± 2.5%) was not statistically higher than that obtained between M-waves in the lateral columns (3.1 ± 2.7%; *P* = 0.22; *N* = 6 subjects; Fig. 7*C*).

## DISCUSSION

In this study, we presented and tested a new system of electrodes transparent to US; i.e., US-EMG electrodes. We specifically evaluated whether surface EMG and US images of high quality could be detected simultaneously from the same portion of the MG muscle with such a system. The noise and the impedance of the electrode-skin interface and changes in image characteristics and in the waveform of surface potentials were quantified to characterize our new electrodes. As discussed below, crucial features of EMGs and US images recorded with the US-EMG electrodes are comparable to those recorded by conventional means.

### Characterization of the US-EMG Electrode-skin Interface

Compared with conventional electrodes, the US-EMG electrodes presented similar electrical noise and impedance values in the electrode-skin interface. Two relevant considerations regarding our noise and impedance measurements prompt from results. First, although the size of the contact surface between the skin and the conductive paste was similar for the two electrode types (see methods), the contact area between metallic material and conductive paste was smaller for the US-EMG electrodes (1 mm^2^) than for the conventional electrodes we used for testing (3 mm^2^). Notwithstanding their different metal-paste contact size, conventional and US-EMG electrodes provided similar noise and impedance values (Fig. 4). Results shown in Fig. 4 suggest that noise and impedance are thus not affected by geometrical characteristics of the electrical contact between the metallic surface and the conductive paste. Second, our measurements provided impedance magnitudes higher than those previously reported in the literature (3, 20). One possible reason for such discrepancy is the number of electrodes across which electrical current was applied. Considering that we measured the impedance between two electrodes, our measurements take into account the impedance of two electrode-skin systems, as well as of the tissue between these electrodes. Moreover, it must be noted that changes in the electrode-skin impedance are due to several factors (23), such as the following: *1*) the skin treatment; *2*) the use and the characteristics of conductive substances; *3*) the frequency band over which measurements are made; and *4*) electrode size and others. Although the lack of standards guiding impedance measurements for EMG systems hampers comparisons between studies, the electrode-skin interface of commercially available and of our US-EMG electrodes had similar characteristics.

### Did the US-EMG Electrodes Affect the Quality of EMGs and US Images?

The use of our US-EMG electrodes provided high-quality surface EMGs with negligible interference from the US probe. During the electrically induced contractions, we secured the US probe over the US-EMG grid of electrodes (Fig. 3*B*) with elasticized bandage. Due to deformation of the matrix material, it is possible that electrodes beneath the US probe (central columns: *columns 2* and *3*) were pressed more strongly against the skin than the lateral columns of electrodes. For this reason, we would expect to observe a greater number of low-quality EMGs detected by central rather than by lateral electrodes. Empirically, though, this hypothesis was not verified. The likelihood of observing missing contacts, short circuits, power line interference, and movement artifacts in the surface EMGs did not depend on whether recordings were taken from electrodes beneath the US probe or not. Furthermore, regardless of whether considering the central or the lateral columns of electrodes, M-waves detected with and without the US probe lying over the gird of electrodes were not statistically different (Fig. 7). Therefore, the surface EMGs recorded with our US-EMG electrodes were consistently insensitive to the presence of US probe and to the inhomogeneous distribution of pressure over the skin, a typical consequence of having to secure US probes on specific skin regions.

The quality of US images was not affected by the use of our US-EMG electrodes. Presumably due to its low acoustic impedance and its acoustic coupling with the skin, the US-EMG electrode system resulted in negligible reflection at the skin interface. The high quality of US images was verified by the statistical agreement between sinusoidal movements of a spring estimated with and without the interposition of US-EMG material between the US probe and the plastic beaker (Figs. 3, *A* and *C*, and 5). Even though the accuracy of tracked oscillations significantly decreased with the frequency or with the oscillation amplitude (Table 1), it did not depend on the use of the US-EMG material. This frequency-amplitude-dependent error between imposed and tracked oscillations must be due to sources other than the US transparent material. Some possible sources of variability could relate to the tracking algorithm and/or potential misalignment between the US probe and the longitudinal axis of the spring.

Similarly to the in vitro testing, the interposition of the US-EMG electrodes between the skin and the US probe did not affect the tracking of gastrocnemius twitches or change in thickness. For all participants tested, movements of gastrocnemius fascicles were tracked remarkably well in US images recorded with the US probe lying over the US-EMG electrodes or directly on the skin. Although the twitches tracked in both conditions had similar shape, as evidenced from the statistically significant correlations reported in Fig. 6, the amount of estimated muscle shortening and lengthening between conditions was not equal across participants. Specifically, with the exception of few cases, twitches tracked with the US probe over the US-EMG electrodes were either smaller or bigger than those tracked with US probe directly on the skin (Fig. 6). The degree of variation was higher for measures of muscle thickness than for length change. Given that imposed changes in spring length were estimated equally well with and without the US-EMG material, we believe the different amounts of gastrocnemius movement estimated across most participants were not due to the US-EMG electrodes. As shown in Fig. 7, changes in M-wave shape were markedly small, suggesting differences in MG twitches between trials were unlikely due to changes in relative position of stimulation electrode and nerve. This discrepancy in estimated muscle movement might have been due to the fact that acquisition of US images in the two experimental conditions required repositioning the US probe over the muscle. Therefore, it is unlikely we recorded US images from exactly the same gastrocnemius region, thus resulting in twitches with similar shapes (i.e., high correlation coefficients) and different amplitudes (slope values lower or greater than one). More significantly, maintaining the same pressure during reattachment of the probe over the muscle with elasticated bandage was difficult. Differences in pressure have been shown to significantly affect both muscle thickness and fascicle length change (44) and are, therefore, likely to be a significant contributor to the variation seen in the results here. Finally, it should also be considered that repositioning the probe required moving the participant, potentially resulting in slightly different postures between trials, which may also have influenced the resultant muscle tissue displacements recorded.

### Featuring US-EMG Electrodes in Different Configurations

The technology we employed in the present study can be easily extended for the design of US-EMG electrodes in different configurations. Our detection system currently consists of 32 electrodes disposed into a grid of 8 rows and 4 columns, with 1 cm center-to-center distance. This electrode configuration was thought to span most of the skin surface covering the large gastrocnemius medialis muscle. Depending on the geometry of the muscle studied, electrodes might be rearranged in different configurations and dimensions. Differently from other materials typically used to build electrode grids [e.g., cushion of plastic foam (3) or flexible printed circuits (20)], the silicon rubber material used to design our US-EMG electrodes allows for bending simultaneously in the transverse and longitudinal direction. Such flexibility further increases the capability of the detection system to adapt to specific muscle morphologies while ensuring the contact between electrodes and skin.

### The Physiological Significance of Simultaneously Detecting EMGs and US Images From the Same Muscle Portion

Currently, simultaneous recordings of US images and surface EMGs are made from adjacent muscle regions. For small muscles such as those in the hand, placing surface electrodes alongside US probes is overtly not viable; in these conditions, EMGs and US images will likely reflect distinct physiological and/or geometrical events. For large muscles such as the MG, surface electrodes and US probes placed alongside each other might sample from the same muscle, although not from the same muscle portion. Whether the consequences of muscle activation are represented equally in EMGs and US images taken from different muscle regions remains unknown. If either activation or tissue displacement distributes inhomogeneously across the muscle volume, it is possible that surface EMGs and US images, recorded from different muscle regions, will reflect different physiological events. Evidence from EMG, at least, has shown local changes in muscle activation in response to fatigue (11, 14), to changes in force direction, to electrically elicited contractions (15, 26), to electrically induced muscle cramps (27) and during human quiet standing (41, 42). Differently from conventional systems of electrodes, our detection system lays a foundation for a new field of integrated investigation of electrophysiological and tissue displacement characteristics.

This US-EMG system could further contribute to the study of neuromuscular physiology. Currently it is not possible to noninvasively study the spatio-temporal propagation of motor output, from motor unit activation to mechanical muscle tissue strain. High-density surface EMG provides a high spatio-temporal resolution of superficial muscles. Thus, generally, the sampling of motor unit activity deep within the muscle (e.g., muscles pinnate in depth direction) or the activity of deep motor units (e.g., parallel-fibered muscles) is not viable. US provides a planar view broadly perpendicular to the surface and thus provides depth information to the spatio-temporal propagation of mechanical tissue strain. Muscle tissue tends to average the effect of motor unit activity from any spatial source, and thus conventional low frame rate US (20–50 frames/s) reveals the integrated effect of regional motor unit activity, but not its temporal propagation in the muscle volume. Instead, high (ultrafast) frame rate US (500–5,000 frames/s) provides temporal resolution equal to that of EMG. These advancements have begun to be used, providing a novel means of investigating electromechanical delay in skeletal muscle (31) and tissue displacement during electrically evoked twitches (7). Such advances in imaging technology are supplemented by the growing number of computer-based image analysis algorithms developed to extract quantitative details of skeletal muscle tissue displacement and fascicle mechanics (e.g., Refs. 4, 5, 22, 29, 34). Our US-EMG electrodes would, for the first time, enable simultaneous assessment of muscle activation and mechanical tissue displacement (see supplemental material; the online version of this article contains supplemental data). In combination with high frame rate US and computational image analysis, our new US-EMG system of electrodes possibly allows the electromechanical imaging of neural output.

The use of US-EMG electrodes might also improve our current understanding of how muscle anatomical features affect the representation of motor units in the surface EMGs. Current knowledge indicates that motor unit action potentials might exhibit different characteristics, depending on the muscle architecture (26) and on the orientation and position of surface electrodes in relation to muscle fibers, innervation zone, and muscle-tendon junctions (12). For instance, theoretical and empirical evidence has shown that, for greater pinnation angles, motor units surface potentials are represented in smaller skin regions (26). Therefore, alterations in the amplitude and bandwidth of surface EMGs with changes in fibers orientation and length might thus be assessed unequivocally with US-EMG electrodes.

A potentially promising clinical application for the US-EMG electrodes might concern the study of fasciculation potentials whose occurrence is often identified from either EMGs or US images (9, 33, 35). Both EMG and US imaging techniques, however, have disadvantages: EMG cannot identify fasciculation occurrences from a large detection volume; by contrast the morphology of the potential cannot be studied through US images. By combining the high sensitivity in detecting fasciculation, provided by US imaging, with the possibility of discriminating fasciculation occurrences in the morphology of surface potentials detected from a large muscle region (9, 19), our US-EMG system of electrodes might contribute markedly to this clinical field of application.

## GRANTS

This study was supported by Compagnia di San Paolo, Fondazione C.R.T., the Fundação de Amparo à Pesquisa do Estado do Rio de Janeiro (INST-110.842/2012), the Wellcome Trust (WT085599MA), and the EPSRC funded Bridging the Gaps: Nano-Info-Bio Project, Grant Reference EP/H000291/1.

## DISCLOSURES

No conflicts of interest, financial or otherwise, are declared by the author(s).

## AUTHOR CONTRIBUTIONS

Author contributions: A.B., T.M.M.V., I.D.L., R.M., and E.F.H.-T. conception and design of research; A.B., T.M.M.V., I.D.L., and E.F.H.-T. performed experiments; A.B., T.M.M.V., and E.F.H.-T. analyzed data; A.B., T.M.M.V., I.D.L., R.M., and E.F.H.-T. interpreted results of experiments; A.B., T.M.M.V., and E.F.H.-T. prepared figures; A.B., T.M.M.V., and E.F.H.-T. drafted manuscript; A.B., T.M.M.V., I.D.L., and E.F.H.-T. edited and revised manuscript; A.B., T.M.M.V., I.D.L., R.M., and E.F.H.-T. approved final version of manuscript.

## Supplementary Material

Supplemental Video
