# Supplementary material for: A novel system of electrodes transparent to ultrasound for simultaneous detection of myoelectric activity and B-mode ultrasound images of skeletal muscles
Source: J Appl Physiol (1985). 2013 Aug 1;115(8):1203–14. doi: 10.1152/japplphysiol.00090.2013 (PMC3798813; doi:10.1152/japplphysiol.00090.2013)
Supplement: Supplemental Video [file supp_115_8_1203__index.html]

A novel system of electrodes transparent to ultrasound for simultaneous detection of myoelectric activity and B-mode ultrasound images of skeletal muscles — Supplemental Video 

# A novel system of electrodes transparent to ultrasound for simultaneous detection of myoelectric activity and B-mode ultrasound images of skeletal muscles

## Supplemental Video

One video in AVI format.

**Files in this Data Supplement:**

- Video S1 - This supplemental video shows the time evolution of activation and architectural changes in the medial gastrocnemius muscle during a single, electrically elicited twitch. The top panel depicts a single M-wave detected by the most lateral and proximal electrode in the grid of 32 electrodes (this M-wave corresponds to that shown in column 1, row 1 in figure 6a). Length change estimated from gastrocnemius in response to electrical stimulation is shown in the bottom panel. Both M-wave and muscle twitch are average across ten consecutive stimuli. The color map shown in the middle panel indicates the amplitude of M-waves detected by each electrode of the EMG-US grid. Such EMG image provides a clear view of the surface distribution of muscle activation in correspondence of the depth view of muscle tissue provided by the US images. The ultrasound video shows the resulting muscle twitch. The image is overlaid with a graphics array with the colours representing the movement velocity of recognisable features tracked within the image. Recognisable features were identified mathematically using methods outlined previously (Darby et al. 2012). Their positions were tracked across images and associated velocity calculated from displacement and time between images. For each image shown in the middle panel, greater M-wave amplitudes and faster movements are indicated by warmer (red) colours, whereas cooler (blue) colours indicate lower muscle activation and small-no movements. US and EMG frames correspond to time instants indicated by the cyan bars.
